# Effect of simulated aging on the color stability and fracture resistance of lithium disilicate laminate veneers with different preparation designs

**DOI:** 10.1186/s12903-026-07883-w

**Published:** 2026-03-04

**Authors:** Amr Rizk, Nouran Mahmoud, Ahmed Abdou, Sarah Omar

**Affiliations:** 1 Prosthetic Dentistry Department, Fixed Prosthodontics Division, Faculty of Dentistry, King Salman International University, El-Tur, South Sinai Egypt; 2 Department of Prosthodontics, Faculty of Oral and Dental Medicine, Badr University in Cairo, Cairo, Egypt; 3 Department of Restorative Dentistry, Faculty of Dentistry, Universiti Malaya, Kuala Lumpur, Malaysia

**Keywords:** Color stability, Fracture resistance, Thermal cycling, Laminate veneers, Preparation designs, Advanced lithium disilicate ceramic, Virgilite-containing lithium disilicate ceramic

## Abstract

**Purpose:**

To evaluate and compare the effect of simulated aging on conventional lithium disilicate (LDS) and advanced lithium disilicate (ALD) computer-aided design-computer-aided manufacturing (CAD-CAM) glass ceramics on color stability and fracture resistance utilizing different veneer preparation designs.

**Materials and methods:**

Two prepared typodont maxillary right central incisors were replicated into forty epoxy resin dies, which were divided into 2 groups based on preparation design (*n* = 20): Group butt joint (BJ), and Group incisal overlap (IO). Each group was further subdivided into two equal sub-groups based on ceramic material (*n* = 10): e.max (IPS e.max CAD, Ivoclar Vivadent, Schaan, Liechtenstein), and Tessera (Cerec Tessera, Dentsply Sirona, Hanau, Germany). After cementation of laminate veneers on the epoxy resin dies, color change and fracture resistance were evaluated before and after 10,000 cycles of thermal cycling. Two-way ANOVA was used to compare the materials and design on the mean color change. For fracture resistance, a three-way ANOVA was used to compare the materials, designs, and aging. Pairwise comparison was tested using independent t-test (α = 0.05).

**Results:**

For color change, Two-way ANOVA revealed that different materials, preparation designs and their interaction showed a non-significant effect on the ΔE_00_ (*p* = 0.352, 0.158 and 0.678, respectively). For fracture resistance, different materials, preparation designs, and aging, had a significant effect on fracture resistance at *p* < 0.05. The interaction between all the groups showed a non-significant effect (*p* = 0.918). Before aging, IO showed significantly lower fracture resistance compared to BJ for both e.max and Tessera. A non-significant difference was shown between the tested materials within the same preparation design. After aging, a non-significant difference between all tested groups was shown.

**Conclusions:**

Within the limitations of this in vitro study, it was concluded that thermal cycling affected both color stability and fracture resistance of both tested materials. The color change for BJ for both materials was within the clinically accepted range. Both tested materials showed comparable mean fracture resistance before and after thermal cycling within the same preparation design. BJ showed significantly higher fracture resistance than IO before thermal cycling; however, the difference between the two tested preparation designs after thermal cycling was not significant.

**Clinical significance:**

Both materials (LDS and ALD) are clinically viable; however, preparation design has a greater impact on mechanical performance than on color stability. BJ performed better than IO for veneer preparation design.

## Introduction

Veneers made of ceramics are widely recommended as the most restorative procedure utilizing less tooth structure and a biocompatible approach for meeting patients’ esthetic needs. They can be helpful for the repair of worn or fractured teeth, for changing the color of teeth, and even changing teeth with misalignment and malformation. This is due to the more recent generations of ceramics and adhesive materials and methods that led to improvement in the mechanical properties while maintaining excellent esthetics reproduction [1, 2].

Because glass ceramics have high translucency and can be produced using a variety of techniques, interest in these materials has grown, leading to the development of various types of all-ceramic systems [3, 4]. Glass ceramics had a better predictive survival percentage (94%) than feldspathic porcelain (87%). Because of its distinct structure and crystal shape, sufficient high fracture resistance, range of color shades, and high translucency, IPS e.max ceramic is frequently utilized for veneers in anterior teeth [5, 6].

In 2006, IPS e.max CAD lithium disilicate was released with a flexural strength of greater than 350 MPa and a noticeably higher fracture resistance in comparison to earlier adhesive glass ceramics [5, 6]. On the other hand, Tessera, a CAD-CAM ceramic, was released as an advanced lithium disilicate by Dentsply Sirona in 2021. The main characteristic that sets this ceramic apart is how fast it can be fired. The unique composition of the ceramic, which is made up of Virgilite (lithium aluminum silicate) and lithium disilicate, allows for a fast firing time; only 4 minutes and 30 seconds are needed for the glaze firing. It is recommended for veneers, inlays, onlays, and crowns in the anterior and posterior regions [6–9].

Regarding the design used for anterior veneers, there is no clinical agreement. The four common incisal preparations are the window, feather edge, incisal overlap (or palatal chamfer), and butt joint (or incisal bevel) [9–13]. Butt joint design has the advantage of easy cementation and control over incisal aesthetics; on the other hand, they are less retentive. Incisal overlap preparation has the benefit of preventing labial displacement, but it also has the drawback of a greater reduction of tooth structure [2, 14–18]. Incisal overlap restorations with a palatal chamfer finish line have only one path of insertion, which helps in preventing misplacement of the restoration during cementation, whereas butt joint restorations have multiple paths of insertion [5, 14]. The failure risk between the incisal overlap type and the butt joint type is unknown [2]. According to certain theories, the incisal overlap preparation eliminates a sharp angle that could encourage the spread of cracks and enhances the surface area for bonding. Moreover, the incisal edge will have an adequate amount of ceramic thickness with incisal overlap preparation [19–22].

The accomplishment of the three primary requirements of strength, fit, and aesthetics determines the success and durability of any intraoral restoration. When choosing a material for tooth restoration, patients’ expectations and needs for aesthetics play a major role, along with new material options and manufacturing processes [23]. Even though ceramics are color stable, internal or external variables may cause aesthetic restorations made of ceramics to discolor. Changes inside the substance itself are considered intrinsic variables, whilst the sticking or uptake of stains from the oral cavity is considered an extrinsic factor [23]. Furthermore, color stability has been demonstrated in literature to be influenced by different kinds of parameters, including the type and shade of the restorative material, the duration and intensity of exposure to staining agents, the consumption of certain foods and beverages, fabrication techniques, finishing methods, the thickness of ceramics, the resin cement, and the polymerization method [5, 24–27].

As color stability is a crucial criterion for the success of aesthetic materials, color measurement tools like spectrophotometers and colorimeters have gained popularity in the assessment of color variations. They provide numerical expressions of color, standardization, and precision. Three-dimensional colorimetric measurements are used in the CIE L*a*b* system to provide data: L* measures color brightness, a* measures red-green content, and b* measures yellow-blue content. Next, L* a* b* is used to determine the color changes (∆E*ab) which, if larger than 3.5 units, will be considered clinically inappropriate [28]. To improve the correction between computed and perceived color differences, it was suggested to use the CIEDE 2000 color difference formula (ΔE_00_) [29]. The ΔE_00_ formula was proven to increase the perceptibility and acceptability of color differences in oral circumstances and more accurately represents how people perceive color differences than the ΔE*ab formula [30].

Defining characteristics like durability and fracture resistance have a significant impact on ceramic materials’ applications because every material has a unique composition and set of qualities. This is especially important because fracture resistance is an essential factor in determining which material will provide the optimum distribution of stress [3]. The three main causes of porcelain veneer failure are microleakage, debonding, and fracture [31, 32]. According to an investigation [32], after an observation of 15 years of clinical service, fractures were the main laminate veneer failure in 67% of overall cases.

The challenging oral environment that restorative materials must tolerate varies depending on the patient. Uncontrollable factors that may affect the longevity of materials include masticatory stresses, occlusal bad habits, nutritional considerations, moisture, and fluctuation of temperature. In vitro simulations can be used to estimate how long dental materials will last, assessing both the mechanical and structural degradation during their performance clinically. In vitro evaluation tests in the laboratory can reproduce the oral cavity variables to some extent [33–37]. Regardless of the contributing factor, whether breathing, food or drink consumption, teeth and dental restorations are constantly subjected to temperature changes. These fluctuations create thermal stress on both natural teeth and dental restorations [8, 38, 39].

Thermal cycling has been widely used in dental studies to replicate the thermal changes that occur in the oral cavity [40]. Aging using thermal cycling illustrates the relationship between the restorative material’s coefficient of thermal expansion and the tooth by simulating the entry of hot and cold items into the oral cavity through the use of temperature parameters [33, 34].

One of the predisposing factors linked to anterior ceramic veneer fracture is the type of ceramic material used and the design of the incisal preparation. Furthermore, intraoral temperature may have an impact on the color stability and strength of ceramics. Considering the multifactorial nature of veneer performance, where color stability and fracture resistance are influenced by material composition, preparation design, and exposure to thermal stresses, an in vitro evaluation under simulated aging conditions becomes essential. Therefore, the aim of this study was to evaluate the effect of thermal cycling on the color stability and fracture resistance of IPS e.max CAD and the Tessera advanced lithium disilicate CAD-CAM blocks. In addition, the study aimed to investigate the incisal preparation design impact (butt joint vs. incisal overlap) on fracture resistance of veneers before and after simulated aging. The null hypotheses tested were: (1) There would be no statistically significant differences in color stability or fracture resistance between e.max and Tessera for each preparation design before and after thermal cycling; (2) There would be no statistically significant differences in color stability or fracture resistance between butt joint and incisal overlap preparation designs for each material, before and after thermal cycling.

## Materials and methods

Sample size estimation was done using a-priori power analysis performed with G*Power (version 3.1; Heinrich Heine University Düsseldorf, Düsseldorf, Germany) indicated that five samples per group would provide 80% power (α = 0.05) to detect a mean difference of fracture resistance of 68 N (d = 2.33) based on parameters observed in a pilot study. This pilot study compared two groups with distinct preparation designs, yielding mean values of 243 N and 311 N (Standard Deviation [SD]: 10.1 and 40.0, respectively) prior to thermocycling. Upon completion of the study, a post-hoc power analysis was performed to validate the full experimental design.

In a 2.5 cm in height by 2 cm in diameter special molds, two typodont right maxillary central incisor teeth (NISSIN Dental Model, Koyoto, Japan) were positioned. To guarantee vertical alignment, a dental surveyor (Ney Surveyor; Dentsply International, PA, USA) was used to help set each typodont tooth in acrylic resin (self-cure acrylic resin, Acrostone, Egypt), in the center of the mold. This was accomplished by applying sticky wax to central portion of the incisal edge and attach it to the surveyor’s analyzing rod along its long axis. The rod was then fastened to the surveyor’s vertically moving arm. After the tooth’s axis was properly positioned, acrylic resin was mixed and added to the mold up to 2 mm below the cemento-enamel junction (CEJ).

The typodonts were scanned using an intra-oral scanner (Primescan, Dentsply Sirona, NC, USA) before the teeth were prepared. A biogeneric copy of the teeth was then stored to be used as a guide for designing standardized restoration. Before the typodonts were prepared, silicone putty impressions (Peakosil Start Kit+, Neosil, Korea) were used to create templates for evaluating the amount of tooth reduction. Veneers preparation kit (Porcelain Veneer Kit, Shofu Inc., Kyoto, Japan) was used to make standard preparations on both typodont teeth. A single experienced operator performed veneer preparations.

Control over the preparation thickness was achieved by using a diamond depth cutting stone. A labial reduction of 0.5 mm that followed the labial surface anatomy in two planes was done using a tapered stone with a round end. With a chamfer finish line of 0.5 mm width, the preparation was stopped proximally just after the proximal line angles and cervically 0.5 mm coronal to the CEJ.

**Fig. 1:**
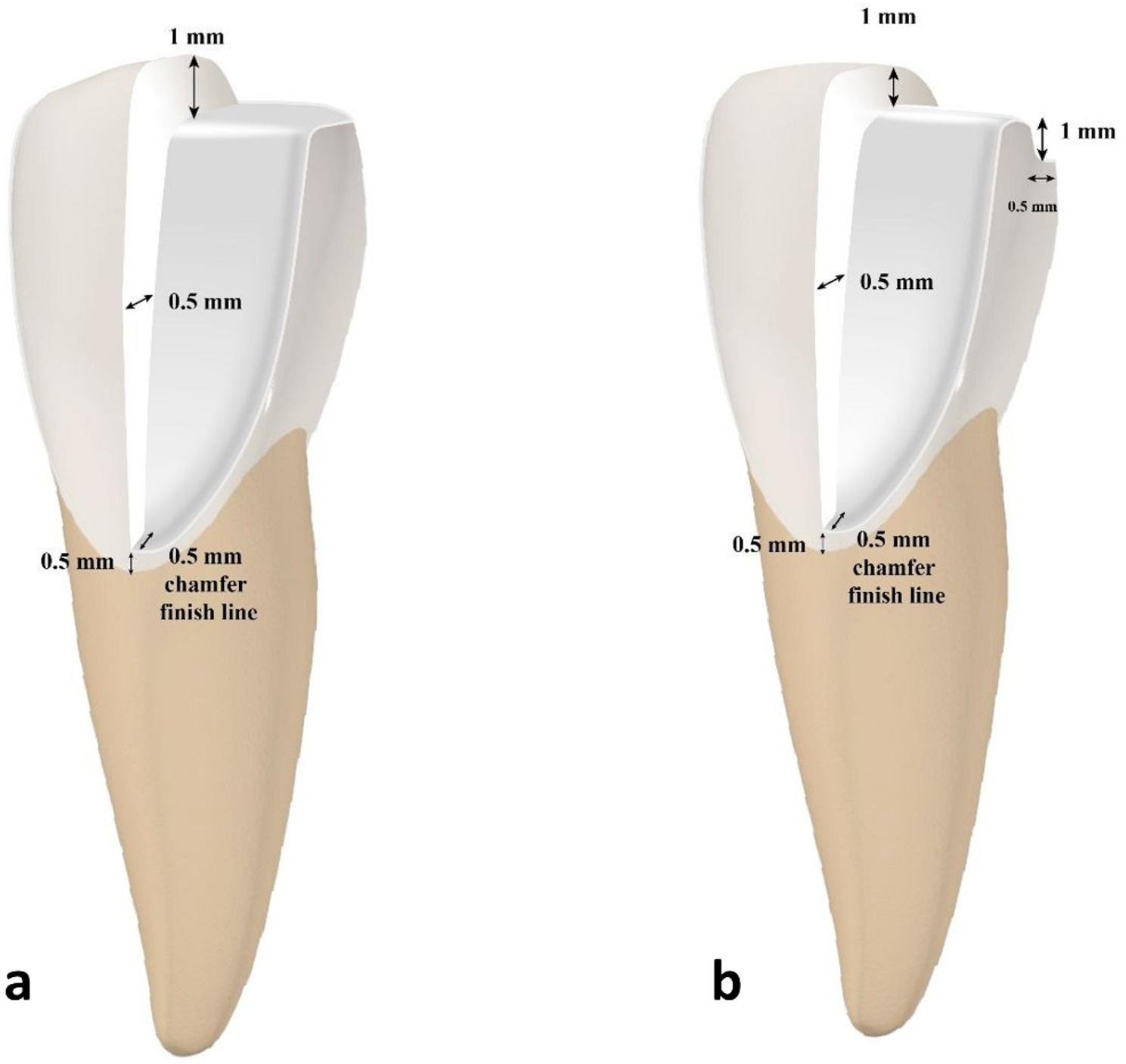
Diagrammatic illustration of the design of preparation and veneers. (**a)** Butt joint design (BJ), (**b**) Incisal overlap design (IO)

A butt joint (BJ) incisal preparation was performed on one tooth by reducing the incisal edge by 1 mm without creating a palatal chamfer. The incisal edge of the other tooth was reduced using an incisal overlap (IO) design, which involved a 1 mm incisal reduction and an extension of the preparation by 1 mm along the palatal wall, terminating with a chamfer finish line of 0.5 mm width. Any sharp angles were rounded to avoid stress concentration **(**Fig. 1**)**. The final step involved duplicating every prepared tooth into 20 epoxy resin dies (Kemapoxy 150 3D, CMB chemicals, Cairo, Egypt).

Typodonts were scanned once again for the designing phase following teeth preparation. An expert CAD-CAM designer started the designing process once a 3D optical picture was imported into the CAD program (CEREC 5.2, Dentsply Sirona, NC, USA). On the digital models, the preparation finish lines were automatically identified and marked. A spacer thickness of 30 μm [41] was designed for the veneers. Using the previously stored biogeneric copy, restoration anatomy was adjusted to bring the teeth back to their pre-reduction dimensions. The thickness of the veneer was examined and confirmed using CAD software prior to starting the milling procedure. The milling procedure started using a wet milling protocol after a CAD-CAM block was fixed and secured into the milling chamber of the milling machine (CEREC MC XL, Dentsply Sirona, NC, USA).

After milling the veneers, the thickness of each veneer was verified using a gauge caliper (Iwanson Dental Gauge Caliper, Waldent, Jalandhar, India). All the milled veneers were examined for any mill-related flaws, defects, cracks, or chipped areas, and then by using magnifying loups (Univet, Rezzato, Italy) with a 3x magnification and optimal lighting, they were tried for their fit onto their corresponding dies. Each veneer was finished and glazed in accordance with the guidelines provided by the manufacturer.

Fitting surfaces for laminate veneers were thoroughly cleaned in an ultrasonic cleaner, then etched for 20 s using a 9% hydrofluoric acid gel (Porcelain Etch & Silane, Ultradent, UT, USA), rinsed for a further 20 s under running water, dried with an oil-free air syringe, and after that a silane coupling agent (Porcelain Etch & Silane, Ultradent, UT, USA) was applied and allowed to dry for a minute. After applying and air-thinning a light-cured dental adhesive bonding agent (All-Bond Universal, Bisco, IL, USA) to the fitting surface in accordance with the manufacturer’s instructions, the bonding agent was light-cured for 10 s. The epoxy dies underwent the following steps: they were cleaned with water, dried with oil-free air, etched for 15 s using 35% phosphoric acid (SELECT HV ETCH, Bisco, IL, USA), and then rinsed with water and dried with oil-free air for ten seconds [42, 43]. Using a micro-brush, two separate coats of the light-cured dental adhesive bonding agent (All-Bond Universal, Bisco, IL, USA) were applied to the preparation by scrubbing for 10 to 15 s per coat without light-curing between coats. After using an air syringe to evaporate the excess solvent for at least 10 s, the bonding agent was cured for 10 s. Each veneer’s fitting surface received a sufficient amount of resin cement (Mojo Veneer Cement, Pentron Clinical, CA, USA), and the veneer was then placed on its corresponding die with finger pressure, replicating the clinical scenario. To remove overflowing cement and tack the veneers into place, the resin cement was light-cured for 1 to 2 s. Finally, each surface of the restoration was cured for 20 s. Light curing was performed using an LED light-curing unit (CuringPen-E, Eighteeth, Sifary Medical Technologies, Jiangsu Province, China) with irradiance of 1200 mW/cm². The light output was verified using a radiometer (C10 LED & Halogen Curing Light Meter; Premium Plus Dental Supplies Inc., NY, USA) prior to specimen preparation to ensure accurate and consistent irradiance. To ensure consistent and precise seating, the adaptation of the veneer margins was carefully verified each time using a sharp dental explorer after placement and removal of excess cement. This approach was intended to replicate standard clinical practice while maintaining consistency across specimens.

The forty epoxy resin teeth with the cemented veneers were divided into two groups (*n* = 20) based on the preparation design: BJ: dies with butt joint veneers, and IO: dies with incisal overlap veneers. Based on the ceramic material used to fabricate the veneers, each group was further subdivided into two equal groups (*n* = 10): e.max: veneers fabricated from conventional Lithium disilicate (IPS e.max CAD, Ivoclar Vivadent, Schaan, Liechtenstein), and Tessera: veneers fabricated from advanced Lithium disilicate CAD-CAM Blocks (Tessera, Dentsply Sirona, Hanau, Germany). All specimens were randomly allocated to experimental groups to minimize selection bias. The composition of the ceramic materials was demonstrated in Table 1. Then each ceramic material group was subdivided into two groups (*n* = 5) based on whether thermal cycling was performed or not. All specimens were stored in distilled water for 24 h before testing.

**Table 1:** Composition of the materials used

Material	Composition	Manufacturer	Translucent/Shade	Lot. number
IPS e.max CAD	70% fine-grain lithium disilicate crystals (Li_2_Si_2_O_5_) in a glassy matrix	Ivoclar Vivadent, Schaan, Liechtenstein	HT/ A2	L18621
Cerec Tessera (Advanced Lithium Disilicate)	Lithium disilicate (Li_2_Si_2_O_5_), and Virgilite (Li_0_._5_Al_0_._5_Si_2_._5_O_6_), which is a Lithium Aluminum Silicate (LAS) both crystals 5 μm in size embedded in glassy matrix with zirconia	Dentsply Sirona, Hanau, Germany	HT/A2	16007898

Specimens designated for simulated aging were subjected to thermal cycling (SD Mechatronik Thermocycler, Germany) to create thermal stresses in the veneers. The specimens were submerged in a bath of distilled water at 5 °C and 55 °C with a dwell time of 20 s and a lag time of 10 s for 10,000 cycles. Color measurement and fracture resistance evaluation tests were performed on the veneers before and after thermal cycling.

A spectrophotometer was used to assess the baseline colors of each specimen (VITA Easyshade ^®^ Advance 4.0, Ivoclar Vivadent, AG, Schaan, Liechtenstein) [44]. using the CIELAB scale and L*, a*, b*. Under the same lighting circumstances, the measurements were taken on a white background. The color was measured by placing the Vita Easy shade’s probe tip on the veneer at the center. Following the gathering of color data for each group, the spectrophotometer was recalibrated. In the calibration part, the tip of the probe was securely positioned and maintained there until the calibration was finished by hearing a beep sound. Using the same spectrophotometer, each specimen’s color values were measured following thermal cycling, and the CIEDE2000 (ΔE_00_) color difference of each specimen was calculated using the following formula [45]:

$$\:{\varDelta\:E}_{00}={\left[{\left(\frac{\varDelta\:L{\prime\:}}{{K}_{L}{S}_{L}}\right)}^{2}+{\left(\frac{\varDelta\:C{\prime\:}}{{K}_{C}{S}_{C}}\right)}^{2}+{\left(\frac{\varDelta\:H{\prime\:}}{{K}_{H}{S}_{H}}\right)}^{2}+{R}_{T}\left(\frac{\varDelta\:C{\prime\:}}{{K}_{C}{S}_{C}}\right)\left(\frac{\varDelta\:H{\prime\:}}{{K}_{H}{S}_{H}}\right)\right]}^{\frac{1}{2}}$$

The differences in lightness, chroma, and hue for a pair of specimens in CIEDE2000 are denoted as *ΔL’*,* ΔC’*,* and ΔH’*, respectively. The rotation function known as *R*_*T*_ accounts for the interaction between hue and chroma differences in the blue region.

The parametric factors *K*_*L*_, *K*_*C*_, and *K*_*H*_ are correction terms for experimental conditions, while the weighting functions *S*_*L*_, *S*_*C*_, and *S*_*H*_ adjust the overall color difference for variations in the position of the color difference pair in *L’*,* a’*, and *b’* coordinates.

**Fig. 2:**
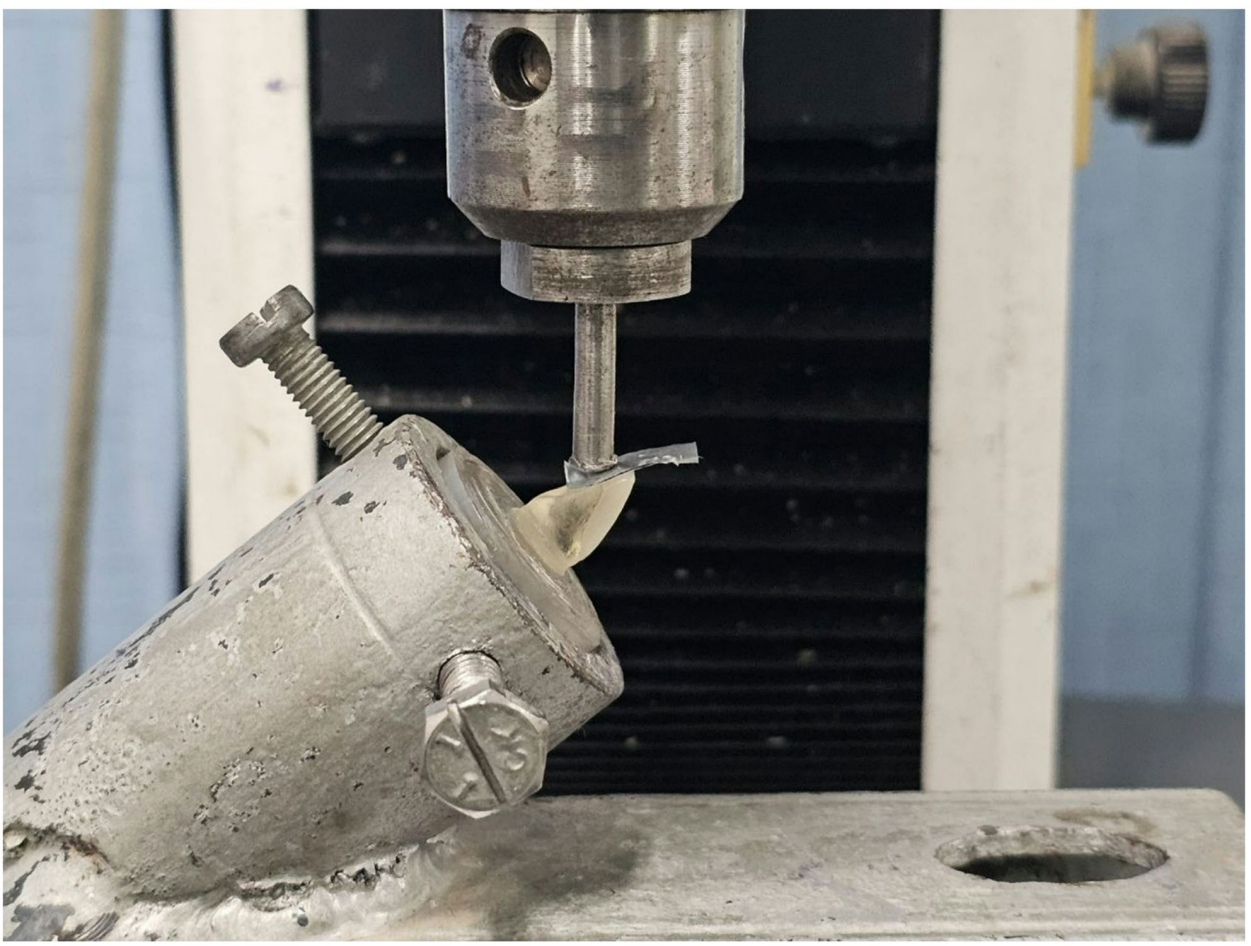
Representative image showing the rod position during the fracture resistance test. *The rod positioned palatally with a layer of tin foil in between restoration and rod*

Specimens were mounted one at a time using a material testing unit controlled by a computer (Model 3345; Instron Industrial Products, Norwood, MA, USA) equipped with a 5 kN load cell. Software (Bluehill Lite software, Instron, MA, USA) was used to record the data. Screws were tightened to firmly attach specimens to the testing machine’s lower fixed compartment. The fracture test was conducted using a flat-tip metallic rod (5 mm diameter) connected to the testing unit’s upper movable compartment, moving at a crosshead speed of 1 mm/min. To ensure uniform stress distribution and minimize the transmission of localized force peaks, the rod was positioned on the palatal surface 1 mm from the incisal edge [46], with a layer of tin foil placed between the indenter and the specimen. This site of load application was chosen to simulate the point of contact where the lower incisors normally engage the upper anterior teeth during protrusive movements, causing lateral stresses, enhancing the clinical relevance of the test. A compressive load was applied at a 135° angle using a 45° angled jig to stabilize the specimen (Fig. 2). Fracture was identified by an audible crack, which was further confirmed by a sudden drop in the load–deflection curve recorded by the software.

A USB Digital microscope (U500X Capture Digital Microscope, Guangdong, China) with an integrated camera was used to take pictures of each specimen for failure mode analysis. The microscope was connected to an IBM-compatible PC at a fixed 10x magnification. One of the following criteria was used to sort the failure modes: adhesive failure (debonding of veneer), cohesive (veneer crack or fracture), mixed (adhesive and cohesive failure), and veneer and die fracture [15].

All collected numerical data were explored for normality using Shapiro-Wilk tests. Two-way ANOVA was used to compare the materials and preparation design on the mean color change. For fracture resistance, a three-way ANOVA was used to compare the materials, preparation design, and aging. Pairwise comparison was tested using an independent t-test. Chi-square test was used to compare failure mood analysis for different tested groups (α = 0.05). All analyses were performed using SPSS version 27 (IBM Corp., NY, USA).

## Results

### Color change

**Fig. 3:**
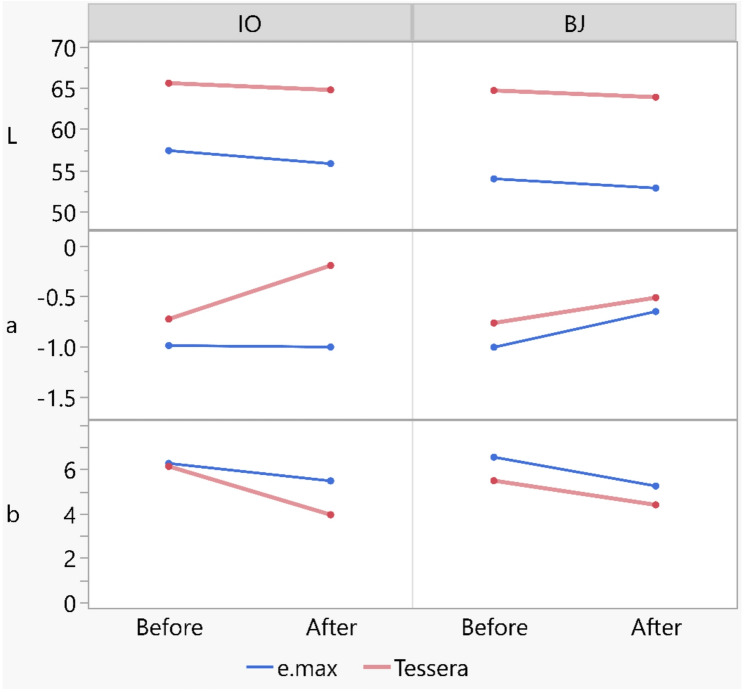
Line chart showing the change in color parameters (**L**, **a**, and **b**) before and after thermal cycling

Two-way ANOVA revealed that different materials, designs and their interaction showed a non-significant effect on the ΔE_00_ (*p* = 0.352, 0.158 and 0.678, respectively) **(**Table 2**)**. The results of ΔE_00_ are shown in Table 3. The change in color parameter is shown in Fig. 3.

**Table 2:** Results of the two-way ANOVA test for the effect of the study variables on color change (ΔE_00_)

Source	Type III Sum of Squares	df	Mean Square	F	Sig.
Materials	0.245	1	0.245	0.888	0.352 ns
Preparation Design	0.574	1	0.574	2.081	0.158 ns
Materials × Preparation Design	0.048	1	0.048	0.175	0.678 ns

*ns* Non-significant

Significance level *p* ≤ 0.05, *significant

**Table 3:** Color change (ΔE_00_) (Mean ± SD [95% CI])

	e.max	Tessera	*p*-value
BJ	1.67 ± 0.38[1.4 to 1.94]	1.75 ± 0.67[1.28 to 2.23]	0.71
IO	1.84 ± 0.63[1.39 to 2.29]	2.06 ± 0.34[1.82 to 2.31]	0.34
p-value	0.47	0.20	

*BJ* Butt Joint, *IO *Incisal overlap

Significance level *p* ≤ 0.05

### Fracture resistance

Three-way ANOVA test results are presented in Table 4. Different materials, preparation designs, and aging showed a significant effect on fracture resistance at *p* < 0.05. The interaction between all the groups showed a non-significant effect (*p* = 0.918).

**Table 4:** Results of the three-way ANOVA test for the effect of the study variables on fracture resistance

Source	Type III Sum of Squares	df	Mean Square	F	*P* value
Aging	26538.465	1	26538.465	10.096	< 0.001 *
Materials	15439.288	1	15439.288	5.874	0.003 *
Preparation Design	1062.785	1	1062.785	0.404	0.021 *
Materials × Aging	36538.263	1	36538.263	13.901	0.529 ns
Aging × Preparation Design	1291.557	1	1291.557	0.491	0.001 *
Materials × Preparation Design	28.061	1	28.061	0.011	0.488 ns
Materials × Preparation Design × Aging	26538.465	1	26538.465	10.096	0.918 ns

*ns* Non-significant

Significance level *p* ≤ 0.05, *significant

Before aging, IO showed a significantly lower fracture resistance compared to BJ for both e.max and Tessera. A non-significant difference was shown between the tested materials within the same preparation design. After aging, a non-significant difference between all tested groups was shown in Table 5. The results of the power analysis (post-hoc) confirm that the completed study was adequately powered for each main effect. Post-hoc calculations showed that the final design achieved 99% power for material type (d = 0.68), 86% for preparation design (d = 0.50) and 100% for aging (d = 1.24).

**Table 5:** Fracture resistance in Newton (N) for all tested groups before and after thermal cycling

Group	Before aging	After aging	*p*-value
e.max-BJ	324.6^ab^±65.9[242.8 to 406.5]	187.2^a^ ± 13.3[170.7 to 203.7]	< 0.001*
e.max-IO	234.6^c^ ± 15.7[215.1 to 254.1]	153^a^±22.9[124.5 to 181.4]	< 0.001*
Tessera-BJ	375.5^a^ ± 42.3[323 to 428.1]	236^a^±107.5[102.5 to 369.4]	< 0.001*
Tessera-IO	266.1^bc^±35.1[222.5 to 309.7]	227.8^a^ ± 34[185.6 to 270.1]	< 0.001*
p-value	< 0.001 *	0.126 ns	

Different letters within each column indicate significant difference

*BJ *Butt Joint, *IO *Incisal overlap

Significance level *p* ≤ 0.05

### Failure mode

**Fig. 4:**
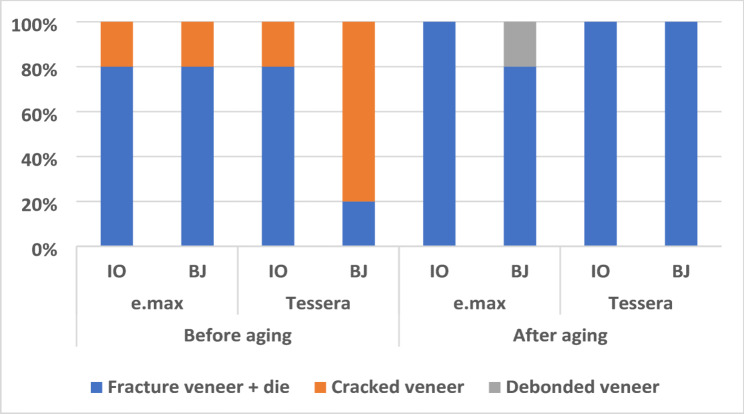
Bar chart showing the Mode of Failure % in each group

**Fig. 5:**
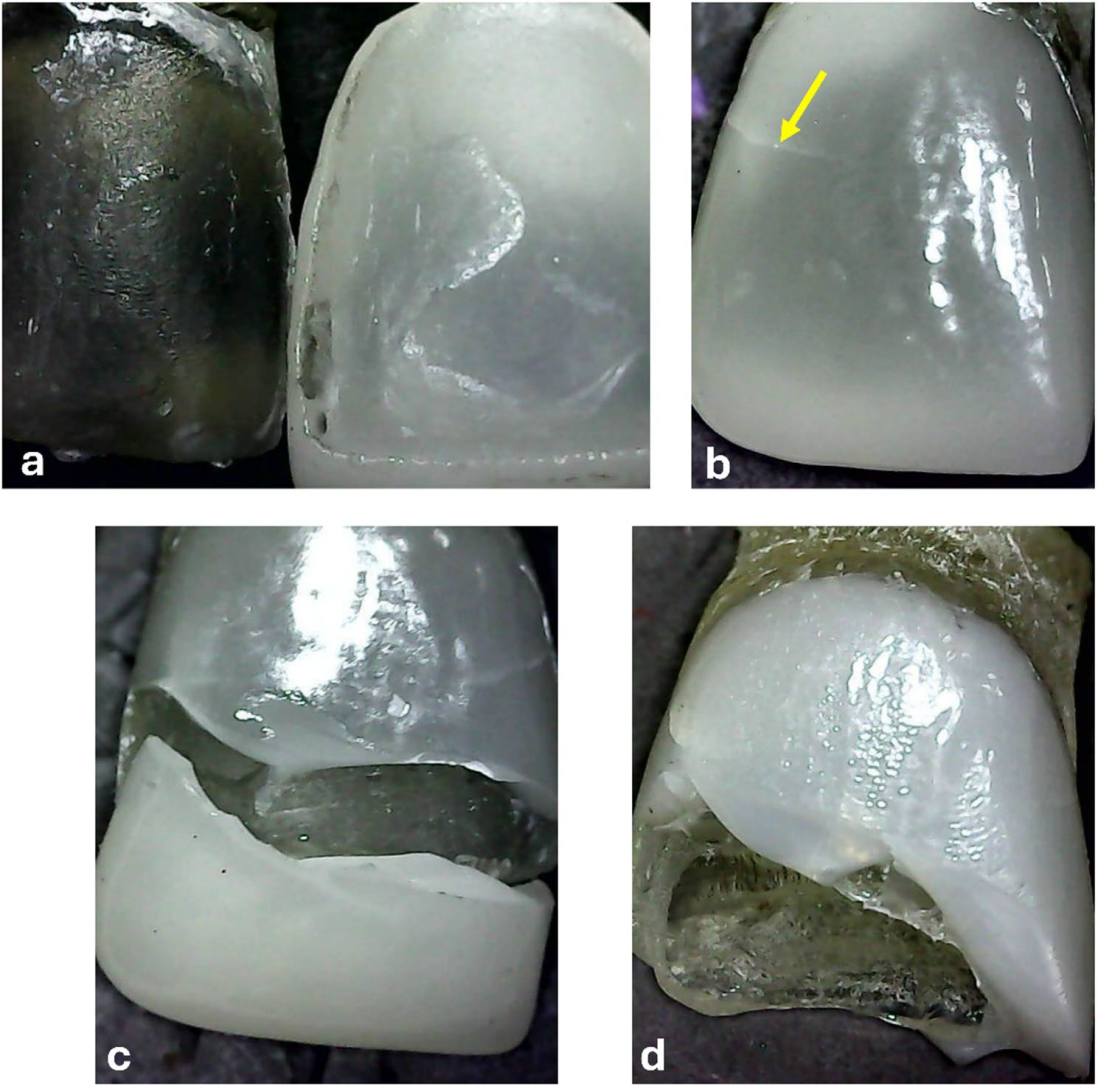
Representative microscopic image of veneers modes of failure. (**a**) adhesive failure (debonding o1.67 ± 0.38). For thef veneer), (**b**) cohesive (veneer crack or fracture), (**c**) mixed (adhesive and cohesive failure), and (**d**) veneer and die fracture

Before aging, fracture of veneer and die occurred in 80% of cases for e.max-BJ, e.max-IO, and Tessera-IO, compared to 20% in the group Tessera-BJ. This difference was statistically significant (*p* = 0.05). However, after aging, no significant difference in failure mode was observed between subgroups (*p* = 0.367). When comparing within the same subgroup before and after aging, only group Tessera-BJ showed a significant difference, with a 100% occurrence of fracture veneer and die after aging compared to 20% before aging (*p* = 0.0009). These findings are detailed in Table 6 & Fig. 4. A representative microscopic image of different failure modes is presented in Fig. 5.

**Table 6:** Descriptive statistics and comparison of fracture mode between different subgroups and within the same subgroup (Chi-square test)

Groups	Preparation	Failure mode	Before aging		After aging		Within group	
			No.	%	No.	%	X^2^	P
e.max	BJ	Fracture veneer + die	4	80	4	80	0.125	0.939 ns
		Cracked veneer	1	20				
		Debonded veneer			1	20		
	IO	Fracture veneer + die	4	80	5	100	1.11	0.29 ns
		Cracked veneer	1	20				
Tessera	BJ	Fracture veneer + die	1	20	5	100	3.75	0.009 *
		Cracked veneer	4	80				
	IO	Fracture veneer + die	4	80	5	100	1.11	0.29 ns
		Cracked veneer	1	20				
Between subgroups	X^2^		3.6		3.15			
	P		0.05 *		0.367 ns			

*ns* Non-significant

Significance level *p* ≤ 0.05, *significant

## Discussion

The impact of artificial aging using thermal cycling on the color stability and fracture resistance of laminate veneers with two designs of preparation constructed from two lithium disilicate ceramic materials was assessed in the current investigation. Based on the results of the present study, the first null hypothesis was accepted as no significant differences were found in color change (ΔE_00_) or fracture resistance between the two materials before and after aging. The second null hypothesis was partially accepted, as there was no difference between the preparation designs in terms of color change or fracture resistance after aging, while BJ demonstrated significantly higher fracture resistance before aging compared to IO.

Results of the present study showed that the highest mean color change value was recorded in Tessera-IO preparation (2.06 ± 0.34), then Tessera-BJ; followed by e.max-IO, with the lowest value recorded in e.max-BJ (1.67 ± 0.38). For the CIEDE 2000 color-difference metric, a ΔE_00_ value greater than 0.8 is generally considered perceptible to the human eye, while a value below 1.8 is deemed clinically acceptable [47, 48]. Accordingly, the color change values reported in this study for IO design of both tested materials were clinically unacceptable.

By simulating the temperature of the oral environment, thermal cycling is a valuable method for expediting artificial aging. Assuming that dental restorations experience twenty temperature fluctuations per day, a year spent in the oral environment is about equal to ten thousand thermal cycling cycles [34, 35].

Generally speaking, thermal cycling aging-related color changes might be caused by the underlying luting agent or the ceramic substance itself. In addition, increased surface roughness, loss of glaze homogeneity, altered translucency characteristics and cracks within the material could be the cause of change in color in the present study [36, 37].

Although there were no statistically significant differences in color change between both materials, the e.max groups demonstrated lower color change value than Tessera groups. This was in accordance with an investigation that examined the impact of resin cements on the translucency and color stability of ceramic laminate veneers made of different ceramic types for diastema closure. The veneers were subjected to a thermo-mechanical aging protocol to replicate one year of clinical service. The lithium disilicate veneers showed the least color change values [25]. This might be attributed to the lithium disilicate color stability and smooth surface after glazing or polishing, as reported in another study [49].

Moreover, a study assessed sectional laminate veneers bonded with two different cement materials and produced with four ceramic materials for color stability. Specimens were cemented and then thermal cycled for five thousand cycles. When comparing veneers of 0.4 mm thickness and cemented with light-curing resin cement before and after thermal cycling, e.max showed more color stability when compared to feldspathic and resin nano-ceramic CAD-CAM blocks. They claimed that since no factor may induce discoloration of the surface of veneer materials, the internal discoloration of the resin luting agent by hydrolysis reaction could be the cause of color change [26].

The results of this study revealed that before aging, the highest mean fracture resistance value was recorded in group Tessera-BJ (375.5 ± 42.3), followed by e.max-BJ, Tessera-IO; with the lowest value recorded in group e.max-IO (234.6 ± 15.7). After aging, however, the highest mean value was recorded in group Tessera-BJ (236 ± 107.5), followed by group Tessera-IO, and e.max-BJ, with the lowest value recorded in group e.max-IO (153 ± 22.9). There was no statistically significant difference between groups.

It has been documented that temperature variations in the oral cavity have a detrimental effect on dental restorations’ strength and hasten the formation of cracks [38]. This effect is due to the ability of water vapour in the surroundings to chemically break strained Si-O bonds, leading to stress corrosion of silicate glasses [39]. The impact of preparation design on the longevity and success of restorations has not received much attention from studies. Whether different tooth preparation designs affect the fracture resistance of ceramic veneers or if one configuration is superior to another is still up for debate.

The current investigation’s evaluation of the influence of tooth preparation design on fracture resistance of laminate veneers made from both tested ceramic materials showed a significant increase in fracture resistance associated with the BJ preparation design before aging. However, after aging, there were no statistically significant differences between different preparation designs of all tested materials. These results could be explained by the fact that BJ design experiences greater compressive stress but less tensile stress than the IO design when evaluated using 2D-finite element analysis (FEA) as reported by a previous study [50]. This results from the more stress concentrated at the restoration interface as a result of the chamfer margin extending to the palatal concavity in the IO design [12]. However, 3D-FEA showed a more uniform stress distribution in the adhesive layer in the IO design than in other designs [13, 51].

After 5,000 cycles of thermal cycling, one study [9] assessed the fracture load of chairside CAD-CAM BJ veneers made from two fully crystallized lithium disilicate ceramic materials (GC Initial LiSi Block and Amber Mill), two pre-crystallised materials (Amber Mill and Tessera) and IPS e.max CAD was used as the control group. Both Tessera and e.max veneers showed fracture resistance values of 503 ± 80 N and 640 ± 109 N, respectively, which were greater than the values obtained in the current investigation. These higher findings could result from a different research design in which the specimens underwent fewer thermal cycling cycles than those used in the current study. Additionally, the materials were tested under a vertical force, which differed from the load application angle used in this investigation.

The results of the current investigation were in contrast with a study that used both in-vitro and FEA studies to assess the fracture load of lithium disilicate glass ceramic (e.max), with two different incisal preparation designs (BJ and IO). They found a significant difference between the tested designs; the IO design withstood stresses better than the BJ design. They related these findings to the fact that the IO design transferred force to hard dental tissue, with the cervical portion of the teeth showing the majority of fractures. Furthermore, they claimed that creating a palatal chamfer also enhanced the size of the veneer, which might lead to a more even distribution of stress [11].

After two years, a clinical study assessed the clinical efficacy of 125 laminate veneers constructed of e.max on twenty-eight patients. The study compared the BJ with IO preparation. Each restoration was checked for debonding and fractures. They found the BJ preparation had a higher survival rate than the IO preparation design, at 94% and 85.7%, although not statistically significant [31].

The findings of the current study were in line with those of Saker S. and Özcan M [15]. and Castelnuovo et al. [16] who reported that, compared with other veneer designs, the BJ design had the highest resistance to fracture. They attributed this high resistance to fracture to a greater risk of fracture in the IO veneers, resulting from stress that is directly over the thin palatal chamfer. They also point out that the BJ design was more convenient for several reasons, including easier tooth reduction, having a facio-palatal path of insertion, the exposed enamel prisms which were more favorable to bonding, ease of impression taking, easy location of the finish line on the model, and elimination of the risk of thin ceramic at the palatal chamfer.

A meta-analysis of in vitro studies [18] found no difference between BJ and IO preparation design in terms of fracture values and patterns. Moreover, other studies [52, 53] reported that the BJ is the most effective preparation for ceramic veneers if incisal coverage is desired.

On the other hand, compared to IO, some studies found that the likelihood of failure is higher for BJ preparation compared to IO [11, 19–22]. These studies attributed the more IO’s favorable results to that the palatal chamfer’s ceramic has been considered as a means of stabilizing the veneer against the mobilization it is subjected to on the facial surface and the absorption of stresses by the cement layer from the area. Moreover, the palatal extension increases the bond surface and provides greater longitudinal exposure of the enamel prisms [19–22]. However, due to the small number of studies and confusing heterogeneity, the failure risk comparison between the BJ and IO designs remains uncertain [2, 12].

Tessera is a pre-crystallized lithium disilicate ceramic composed of approximately 90% lithium disilicate and 5% virgilite embedded in a zirconia-enriched glassy matrix. The lithium disilicate crystals measure approximately 0.5 μm, while virgilite crystals are smaller, around 0.2–0.3 μm [7, 9]. This dual-crystal microstructure, with virgilite formation by a process known as matrix firing, may enhance stress distribution, promote crack deflection, and limit crack propagation, thereby contributing to the obtained study outcome. That unique microstructure helps with the output similarity for the fracture resistance to e.max, particularly after artificial aging.

To the best of the authors’ knowledge, only one previous research [9] has evaluated the fracture resistance of anterior veneers made of Tessera, which can be compared to the results of the present study. Further investigation on the impact of preparation design on the fracture resistance of Tessera veneers may be necessary due to the lack of such knowledge. Also, no previous studies compared BJ and IO veneer preparation restored with both e.max and Tessera in terms of color stability or fracture resistance after simulated aging, making it difficult to directly correlate our results to the co-existing literature.

In the current study, the failure modes were evaluated microscopically using one of the following criteria: adhesive (debonding of veneer), cohesive (veneer crack or fracture), mixed (adhesive and cohesive failure), and veneer and die fracture. Before aging, 80% of e.max-BJ and e.max-IO specimens failed by fracture of veneer and die, while 20% failed by cracking of the veneer (cohesive). Regarding the Tessera, BJ failed by fracture of veneer and die in 20% of the specimens and by cracking of the veneer (cohesive) in 80% of the specimens. In the IO group, 80% failed by fracture of veneer and die, while 20% failed by cracking of the veneer (cohesive). After aging, 80% of the specimens in e.max-BJ failed by fracture of veneer and die and 20% failed by debonding (adhesive). In the e.max-IO group, veneers failed by fracture of veneer and die in 100% of the specimens. Both veneer designs of Tessera failed by fracture of veneer and die in 100% of the specimens. Failure by veneer and die fracture was viewed as the least favorable from a clinical standpoint because it cannot be repaired intraorally due to die fracture.

Two studies [15, 54] reported four modes of failures of veneers made from different ceramic materials. Adhesive failure (debonding of laminate), cohesive (laminate crack or fracture), mixed (adhesive and cohesive failure), and root fracture modes were seen in these studies. According to one study [15], both preparations, the BJ and IO, showed a greater rate of adhesive failure (40%) with veneer fractures. According to another research [20], adhesive failures accounted for 50% of the observed failures in both designs of preparations.

While another investigation [52] showed that cervical fracture was more common in IO preparation (25%) than in BJ preparation (12.50%), coronal fracture was more common in BJ preparation (81.25%) than in IO preparation (62.50%). Oblique, incisal, middle, and cervical third fracture patterns were described by one study [52] where fracture pattern analysis showed that there were more middle-third fractures in both preparations.

The variations in each study related to preparation methods, porcelain type, adhesion resin, and maintenance conditions could account for the disparity in failure mode. Additionally, different mounting devices, loading angles and points, plunger dimensions and shapes, load types (static vs. cyclic), and thermal cycling techniques have all been used in various investigations. Consequently, because of the variability of study design, lack of standardization of loading circumstances, and variable quality of abutment teeth, it is difficult to draw conclusions from these investigations [2, 10, 12].

In the current study, to ensure consistent testing conditions, the study was performed on epoxy resin abutments in order to standardize the size of veneers and because natural teeth exhibit significant differences due to age and individual structure, size, shape, storage conditions of extracted teeth, and the influence of the adhesive bond’s strength on the biomechanical behavior of the porcelain veneer [8, 55].

Due to the limitations of the current study, it should be acknowledged that restoration’s resistance to applied forces is only one aspect of its mechanical performance in the oral environment. Although static load-to-failure testing provides a standardized approach for material comparison and allows evaluation of the tooth-restorative complex’s fracture behavior and estimation of failure risk [2, 56], it does not fully replicate the complex masticatory process. Additionally, in this study, the point of force application was chosen to simulate protrusive interferences rather than normal mastication, which may lead to an underestimation of actual fracture resistance. The sample size was determined a-priori based on a large effect size from a pilot study, and post-hoc power analysis confirmed sufficient statistical power despite the limited number of samples. While in vivo testing remains the most accurate method to assess veneer performance under functional conditions, the multitude of intraoral variables makes it difficult to isolate specific causes of failure [57]. Finally, the use of epoxy resin dies, although advantageous for standardization, does not fully replicate the properties of natural teeth and may have influenced the outcomes, particularly after thermal cycling [8]. Taken together, these considerations highlight that the current results are best interpreted as comparative preclinical data rather than definitive predictions of clinical performance.

It appears that the material used to make ceramic laminates has a significant impact on their long-term prospects. Furthermore, the durability of laminate veneers can be impacted by a number of additional parameters, including the depth of tooth preparation and the thickness of the ceramic [3]. In addition, all processing sequences, adhesive systems, type of material, porcelain etching, light-curing, and resin cements must be carefully considered and managed while working with laminate veneers. It might be advantageous for practitioners to follow every step of the procedure carefully and methodically in order to extend the longevity of restorations in laminates [5].

Further investigations are warranted to enhance the clinical relevance of these findings. Future studies could include the use of extracted human teeth to better mimic natural substrate properties, evaluation under more complex multi-directional loading patterns and occlusal contacts and in vivo clinical trials to validate the comparative performance of different materials and preparation designs. 

## Conclusion

Within the limitations of this in vitro study, it was concluded that:

1. The butt joint preparation design maintained clinically acceptable color after thermal cycling.
2. The butt joint preparation design exhibited higher fracture resistance than the incisal overlap design before thermal cycling, although after thermal cycling, both preparation designs showed comparable fracture resistance values.
3. Conventional LDS and advanced LDS showed comparable fracture resistance following thermal cycling.
4. Thermal cycling reduced both color stability and fracture resistance in the tested materials.

## Data Availability

Dataset used and analyzed data can be available from the corresponding author on reasonable request.
